# Comparing the Effectiveness of Vitamin B6 and Ginger in Treatment of Pregnancy-Induced Nausea and Vomiting

**DOI:** 10.1155/2013/927834

**Published:** 2013-10-22

**Authors:** Ezzatalsadat Haji Seid Javadi, Fatemeh Salehi, Omid Mashrabi

**Affiliations:** ^1^Department of Obstetrics and Gynecology, Qazvin University of Medical Sciences, Qazvin, Iran; ^2^Internal Medicine Department, Faculty of Medicine, Tabriz University of Medical Sciences, Iran

## Abstract

*Objective.* Comparing the effectiveness of vitamin B6 (40 mg twice daily) and ginger (250 mg four times daily) in treatment of pregnancy nausea. *Methods.* In a clinical trial in health centers of Qazvin University of Medical Sciences from November 2010 to February 2011 on pregnant mothers, the effects of vitamin B6 (40 mg twice daily) and ginger (250 mg four times daily) were evaluated in treatment of pregnancy nausea. *Results.* In both groups, treatments with vitamin B6 or ginger led to significant reduction in MPUQE score. Scores of symptoms at the day before treatment in vitamin B6 and ginger groups were 9.35 ± 1.97 and 9.80 ± 2.03, respectively, and reduced to 5.98 ± 1.45 and 6.28 ± 1.63, respectively, in the fourth day of treatment; however, mean changes in the two groups were not significantly different. Mean changes of MPUQE score in ginger and vitamin B6 groups were 8.32 ± 2.19 and 7.77 ± 1.80, respectively, showing no significant difference (*P* = 0.172). *Conclusion.* Vomiting was more reduced in vitamin B6 group; however, this reduction was not statistically significant. There was no significant difference between the two groups in nausea occurrences and their duration. No side effect was observed in either group.

## 1. Introduction

Nausea is the common complaint of women in the first half of pregnancy period, and 85% of pregnant women experience it in early pregnancy [1]. This symptom usually begins between the first and second forgotten menstruations, beats its peak at week 9, and continues until around week 14 to 16 [1]. Nausea is more severe in the morning and may continue throughout the day. 50% of cases spontaneously recover until week 14 and 90% until week 22. At 63% of cases, there is a history of nausea in a previous pregnancy [2]. Characteristics and severity of pregnancy nausea are similar to nausea caused by cancer chemotherapy. Nearly 50% of these people are absent from work for at least one turn [3]. 

Although this symptom gets spontaneously recovered with the time passing, it can place a great stress on the pregnant woman and those around her and disturb her work so that, in 25% of cases, the employed pregnant women often require a leave. This symptom can even lead to depression [4].

Nausea and vomiting are the most common symptoms experienced in early pregnancy, with nausea affecting between 70 and 85% of women. About half of pregnant women experience vomiting [5]. Antiemetic medication appears to reduce the frequency of nausea in early pregnancy [5].

Ginger has been used throughout the world as a therapeutic agent for centuries. The herb is increasingly used in western society also, with one of the most common indications being pregnancy-induced nausea and vomiting (PNV) [6].

The results for the treatment of nausea and vomiting in pregnancy are encouraging; however, ginger should be applied for the time being only in controlled clinical studies [7].

The cause of pregnancy nausea is not yet known. Hence, a wide range of drug groups are experientially prescribed. In early pregnancy, use of chemical drugs is avoided, as far as possible, due to their potential teratogenic effects. So, there is an increasing tendency to alternative therapies. Vitamin B6 is the first-line treatment of pregnancy nausea. Although many pregnant women need additional medications such as antihistamines, these drugs have numerous side effects such as muscle weakness, drowsiness, dry mouth, and visual disturbances. Since a long time ago, ginger has been used as, antinausea an agent in traditional medicine of India, China, and Iran. In different studies, use of ginger has shown no adverse effects on pregnancy outcomes. Ginger has been more effective than placebo in reducing pregnancy nausea. In some studies, effectiveness of ginger has been reported to be equivalent to or more than that of vitamin B6 [8, 9].

The aim of this study is to compare the effectiveness of vitamin B6 (40 mg twice a day) and ginger (250 mg four times a day) in treatment of pregnancy nausea.

## 2. Methods and Materials

In a clinical trial study in health centers of the University of Medical Sciences of Qazvin from November 2010 to February 2011 on pregnant mothers, the effects of vitamin B6 (40 mg twice a day) and ginger (250 mg four times a day) were evaluated in treatment of pregnancy nausea.

According to the conducted studies, the mean and SD of symptom scores after treatment with vitamin B6 were 4.8 ± 2.2. If the new therapy can reduce the mean symptom score by 30%, it appears to be a good alternative [8, 9]. Accordingly, regarding the reliance index of 95% and test power of 80%, the sample size for each group was calculated to be 46.

The population under study consisted of singleton pregnant women with gestational age of less than 17 weeks suffering from pregnancy nausea referred to the health centers of University of Medical Sciences of Qazvin. Patients with a background disease (i.e., urinary tract infection, gastrointestinal, hepatic, biliary, bloodclotting, thyroid, diabetes, or hypertension diseases), taking any kind of drugs, suffering from hyperemesis gravidarum, and food intolerance, with a history of recent hospitalization due to pregnancy-induced nausea, allergenic to ginger, and with twin or molar pregnancies, were excluded from the study. The patients were enrolled in the study after acquiring full knowledge and obtaining written consent and were randomly divided into two treatment groups (vitamin B6 and ginger) and went under treatment with tablets of vitamin B6 40 mg every 12 hours or ginger capsules 250 mg every 6 hours for 4 days. The patients were alerted about the possible side effects such as heartburn and drowsiness to report in case of occurrence. Both groups also got diet improvement.

During treatment, patients in both groups were examined in terms of occurrence times of nausea and the number of dry retches and vomiting. Then, the symptoms of patients were scored by MPUQE scoring system. 

In MPUQE scoring system, times of feeling of nausea during a day, number of occurrences of vomiting during a day, and number of retches during a day were scored on a 1–5 scale, and, accordingly, the score 6 or lower was considered as mild, 7 to 12 as moderate, and 13 or higher as severe symptoms.

## 3. Results

During the study, 102 cases were enrolled into the study, out of which 7 cases (6.8%), including 3 from vitamin B6 group and 4 from ginger group, were excluded.

47 patients were treated with 250 mg ginger capsules every 6 hours and 48 patients with vitamin B6 tablets every 12 hours. The two groups had no difference in terms of the basic variables (age, weight, height, body mass index (BMI), gestational age, parity, gravidity, number of previous miscarriages, and a history of pregnancy-induced nausea) (Table 1).

MPUQE score before and after treatment in both groups is shown in Table 2. The scores of the day before treatment and the first, second, third, and fourth days of treatment were not significantly different in the two groups.

Comparing pre- and posttreatment scores, both treatments applied (with vitamin B6 and ginger) had led to significant reduction in MPUQE score.

The scores of symptoms at the day before treatment in vitamin B6 group and ginger group were 9.35 ± 1.97 and 9.80 ± 2.03, respectively, which, in the fourth day of treatment, had reduced to 5.98 ± 1.45 and 6.28 ± 1.63, respectively; however, the mean changes in the two groups were not significantly different.

The mean change of MPUQE score was 8.32 ± 2.19 in ginger group and 7.77 ± 1.80 in vitamin B6 group which was not significantly different (*P* = 0.172).

Number of retching times in vitamin B6 group was more reduced; however, this reduction was not statistically significant (*P* = 0.333). There was also no significant difference between the two groups in terms of number of occurrence of nausea (*P* = 0.158) and its duration (*P* = 0.148), and no side effect was observed in the two groups. 

## 4. Discussion and Conclusion

Nausea in early pregnancy is still considered as a physical, emotional, social, and economic problem, for which many therapeutic methods have been proposed and applied. Administration of vitamin B6 is the first-line treatment of pregnancy-induced nausea. In the conducted studies, ginger has proven to be more effective than placebo in reducing pregnancy nausea; in some studies, its effect has been reported to be equal to or even more than that of vitamin B6.

In this study, we intended to evaluate and compare the effects of vitamin B6 and ginger in treatment and control of the symptoms of illness in Iranian patients; so that, in case of sufficient effectiveness at least equal as an alternative cure in treatment of our patients.

The use of ginger in early pregnancy will reduce their symptoms to an equivalent extent as vitamin B6 [9].

Results of a study by Portnoi et al. [10] suggest that ginger does not appear to increase the rates of major malformations above the baseline rate of 1% to 3% and that it has a mild effect in the treatment of NVP.

In this study, the two study groups of vitamin B6 and ginger were identical in terms of the basic variables and also admission of people.

Compared to the last day of therapy, the pretreatment score of symptoms in both groups had a significant reduction; however, the changes of the symptoms in the two groups were similar. Mean change was 8.32 ± 2.19 in the ginger group and 7.77 ± 1.80 in vitamin B6 group (*P* = 0.172).

Chittumma et al. [11] treated 126 pregnant women with 650 mg ginger or 25 mg vitamin B6 three times a day for 4 days, where both methods were significantly effective in reducing nausea, retch and vomiting; however, efficiency of ginger was significantly higher than that of vitamin B6. The complications in both groups were reported to be slight and identical.

Ensiyeh and Sakineh [12] treated 70 pregnant women in two equal groups with 1 g ginger or 40 mg vitamin B6 daily for 4 days. In this study, ginger was more effective than vitamin B6 in reducing nausea and vomiting, but both had similarly led to a significant reduction in nausea occurrences.

In these two studies [12, 13] where the effect of ginger has been reported to be higher than that of vitamin B6, where the dosages of vitamin B6 were 75 mg and 40 mg, respectively, which are lower than the dosage applied in our study (80 mg).

Sripramote and Lekhyananda [8] in a clinical trial treated 138 pregnant women with 500 mg ginger or 10 mg vitamin B6 three times a day. They compared the symptoms at the day before treatment with those of the third day of treatment and found that nausea and vomiting occurrences in both groups were significantly reduced; however, there was no significant difference.

Smith et al. [9] in a randomized clinical trial compared the effect of ginger and vitamin B6 in relieving pregnancy-induced nausea and vomiting in 291 pregnant Australian women. These cases were treated with 1 g ginger or 75 mg vitamin B6 for 3 weeks, where ginger has been as effective as vitamin B6 in reducing nausea, retch, and vomiting.

While data are insufficient to recommend ginger universally and there are concerns about product quality due to limited regulation of dietary supplements, ginger appears to be a fairly low-risk and effective treatment for nausea and vomiting associated with pregnancy [13].

In our study, there was no significant difference between effectiveness of ginger and vitamin B6 in reducing the symptoms of pregnancy-induced nausea, and both were similarly effective. Vitamin B6 was more effective in reducing retches; however, this effectiveness was not significant. Both medications were equally effective in reducing occurrence of vomiting and duration of nausea. No side effects were observed in either of the two groups. However, a study with longer treatment duration is recommended to check the possible side effects.

## Figures and Tables

**Table 1:** Evaluation of characteristic variables between the two groups.

Variable	Group*	
	Ginger	Vitamin B6
Age (mean ± SD (year))	26 ± 4.0	27 ± 4.2
Weight (mean ± SD (kg))	61.4 ± 7.2	62.2 ± 6.7
Height (mean ± SD (m))	1.60 ± 0.05	1.58 ± 0.05
BMI (mean ± SD (kg/m^2^))	24.4 ± 3.4	24.3 ± 3.0
Gestational age (mean ± SD (day))	62.9 ± 8.1	62.9 ± 8.6
Gravidity (%)		
1	45.1%	58.9%
2	35.3%	31.4%
3	17.6%	5.9%
4	2%	2%
5	0%	2%
Parity (%)		
0	58.8%	68.8%
1	29.4%	27.5%
2	11.8%	2%
3	0%	2%
Abortion (%)		
0	80.4%	80.4%
1	15.7%	17.6%
2	3.9%	0%
3	0%	2%
History of nausea and vomiting in previous pregnancy		
+	45.1%	37.3%
−	54.9%	62.7%

*Two groups were matched in all variables (*P* > 0.05).

**Table 2:** Nausea and vomiting score (mean ± SD) of patients in two groups before and after treatment.

Group	Before	2 days late	3 days late	4 days late	P*
Ginger (*n* = 47)	9.80 ± 2.03	8.94 ± 2.11	7.43 ± 2.17	6.28 ± 1.63	*P* < 0.001
Vitamin B6 (*n* = 48)	9.35 ± 1.97	8.51 ± 1.98	7.22 ± 1.90	5.98 ± 1.45	*P* < 0.001
*P* ^*¥*^	0.290	0.623	0.351	0.172	—

*Within groups. ^*¥*^Between groups.
